# Effectiveness of a Behavioral Activation Intervention for Peripartum Women with Opioid Use Disorder

**DOI:** 10.1007/s10880-023-09984-y

**Published:** 2024-05-16

**Authors:** Michael R. Vilensky, Nicole A. Arrato, Kristen M. Carpenter

**Affiliations:** 1 Department of Psychiatry and Behavioral Health, The Ohio State University, Columbus, OH USA; 2 Department of Psychology, The Ohio State University, Columbus, OH USA

**Keywords:** Peripartum depression, Anxiety, Opioid use disorder, Integrated care models, Pregnancy

## Abstract

Pregnant women with opioid use disorder show elevated rates of comorbid mental health problems, both of which are associated with negative health outcomes for mothers and children. There is substantial evidence supporting the benefits of treatment of perinatal opioid use disorder, as well as perinatal depression and anxiety, but there are gaps in knowledge about the effectiveness of perinatal behavioral health interventions in the context of co-occurring substance use disorder. The current study seeks to address this gap by examining outcomes of a behavioral activation treatment in a group of peripartum women with opioid use disorder (*N* = 68). Behavioral activation has shown promise in treating co-occurring depression and substance use problems. The intervention was delivered as part of an integrated care treatment model, in which patients received co-located obstetric, substance use, and mental health care in a hospital-based clinic. Hierarchical linear modeling was used to assess change in symptoms over time. Results suggest that the group behavioral activation intervention was associated with reduced depression and anxiety symptoms, demonstrated by significant reductions in PHQ-9 and GAD-7 scores over the course of treatment. Moreover, there were indications that increased attendance was associated with further reductions in depressive symptoms. Results contribute to understanding the effectiveness of behavioral activation in the context of peripartum opioid use disorder. Findings also add to the evidence supporting integrated care models and offer a potential blueprint for improving outcomes and reducing barriers to care in this population.

## Introduction

Among the many damaging effects of the opioid epidemic has been its impact on pregnant women and their children. As the prevalence of opioid use disorder has increased in the population at large, rates have also increased among pregnant women. In the period from 2010 to 2017, the percentage of pregnant women with opioid use disorder at delivery more than doubled, with rates increasing from 3.5 to 8.2 per 1000 deliveries (Hirai et al., 2021). Rates of neonatal opioid withdrawal syndrome (NOWS) have increased correspondingly, from 4.0 to 7.3 infants born with NOWS per 1000 deliveries from 2010 to 2017. In addition to NOWS, opioid use disorder during pregnancy is associated with other negative outcomes, including fetal growth restriction, fetal death, preterm labor, and maternal mortality (Krans & Patrick, 2016; Whiteman et al., 2014). Maternal mortality rates have increased in recent years, with several states reporting overdose as a leading cause of pregnancy-related death (Goldman-Mellor & Margerison, 2019; Smid et al., 2019).

Pregnant women with substance use disorders also show elevated rates of comorbid mental health problems, particularly mood, trauma-related, and anxiety disorders. For pregnant women with opioid use disorder, 25–33% have reported co-occurring depression and anxiety, which is nearly double that of pregnant women without substance-related problems (Arnaudo et al., 2017). The authors note that these rates are likely underestimates, given fears about implications for reporting agencies. The negative consequences of depression and anxiety during pregnancy have been studied extensively. Perinatal depression and anxiety are associated with poorer health-related quality of life and lost productivity (Bauer et al., 2016). Pregnant women with perinatal depression and anxiety are at higher risk for postpartum psychiatric disorders (Robertson et al., 2004). Perinatal depression and anxiety are also associated with negative outcomes for children, including elevated rates of pre-term birth, infant death, teacher-reported special educational needs, emotional problems, and conduct problems (Bauer et al., 2016).

While the negative consequences of perinatal substance use and mental health problems are substantial, research shows that psychiatric and psychosocial treatments can provide significant benefits. Indeed, the American College of Obstetricians and Gynecologists treatment guidelines point toward the importance of linking pregnant women with evidence-based care for opioid use and mental health problems (American College of Obstetricians & Gynecologists, 2017). For pregnant women with opioid use disorder, opioid agonist pharmacotherapy is the first-line treatment and has been shown to be highly effective in reducing relapse rates and ameliorating the negative consequences of opioid use (Klaman et al., 2017). Psychosocial treatments have also been shown to enhance these outcomes when combined with pharmacotherapy (Dugosh et al., 2016). Similarly, psychosocial treatments have shown effectiveness in the treatment of perinatal anxiety and depression. A systematic review of 78 studies showed strong benefits for cognitive behavioral therapy (CBT) and interpersonal therapy (IPT) in reducing mental health symptoms during the perinatal period (Nillni et al., 2018).

Despite the evidence supporting the benefits of treatment of perinatal opioid use disorder, as well as perinatal depression and anxiety, little is known about the benefits of concurrent treatment. That is, existing studies have looked at substance use and mental health treatment independently. In fact, the presence of a substance use disorder has often been an exclusion criterion in evaluations of perinatal mental health interventions. As such, the effectiveness of perinatal mental health treatment in the context of co-occurring substance use treatment is largely unknown. However, there is considerable evidence suggesting that such interventions are crucial for this population. Notably, increased severity of mental health problems is associated with lower adherence to substance use treatment and perinatal obstetric care (Bauer et al., 2016). Perinatal depression and anxiety exacerbate substance use problems and negatively impact obstetric outcomes (Arnaudo et al., 2017). Perinatal psychiatric symptoms also raise risk for maternal death due to overdose (Campbell et al., 2021).

The current study seeks to address this gap in knowledge by examining the outcomes of a behavioral intervention for peripartum women with opioid use disorder. Behavioral activation, an intervention that seeks to improve mood symptoms by enhancing positive reward behaviors, has shown promise as a treatment for co-occurring depression and substance use problems (Martinez-Vispo et al., 2018). Importantly, reward behaviors are central to both depression and substance use. Behavioral theories of depression stress the role of withdrawal from rewarding activities as a key maintenance factor in depression (Dimidjian et al., 2011). That is, when people stop engaging in hobbies, social interactions, and other pleasant activities, they lose touch with naturally occurring positive reinforcers and fall further into depression. In substance use disorders, individuals tend to withdraw from rewarding activities that are not substance-related, resulting in increased reliance on substances (Higgins et al., 2004). Over time, substances crowd out other rewarding activities, distancing people from the positive experiences and support that might help them recover. Given the key role of reward behaviors in depression and substance use, behavioral activation has been increasingly considered as a tool to target both problems simultaneously. Indeed, several studies have demonstrated the effectiveness of behavioral activation for co-occurring depression and substance use outcomes (Martinez-Vispo et al., 2018). To build on this work and examine outcomes in the context of peripartum substance use, the current study utilized a manualized group behavioral activation intervention designed to increase rewarding experiences and reduce avoidant behavior in peripartum women with opioid use disorder (Daughters et al., 2018; Dimidjian et al., 2017). We hypothesized that the group intervention would reduce depression and anxiety symptoms for participants.

Moreover, the intervention’s setting in a real-world, hospital-based clinic allows for expanded exploration of the potential benefits of integrated care, in which obstetric, substance use, and mental health treatment is offered in a co-located setting. Integrated care models aim to reduce barriers to care that predominate among peripartum women with OUD and in need of mental health treatment. Research has demonstrated significant barriers to care, including transportation and financial limitations, waiting lists for services, few clinics willing to treat dual-diagnosis pregnant women, and stigma directed at pregnant women with SUDs (Goodman, 2009). These barriers result in the majority of peripartum women with OUD failing to receive adequate treatment (Raffi et al., 2021). The clinic in which the current intervention was tested seeks to reduce these barriers by providing an integrated care model involving co-located high-risk prenatal care, medication for opioid use disorder, and psychosocial interventions for both substance use and mental health disorders. Recent research has pointed toward enhanced outcomes associated with integrated care models, including lower rates of pre-term delivery, shorter duration of post-delivery hospitalizations for infants, and reduced rates of positive urine toxicology screens at delivery (Goodman et al., 2022). We aim to add to this literature by examining mental health outcomes, which have not been evaluated in previous studies of integrated care models. Additionally, there has been some concern that integrated care settings may overwhelm patients by increasing the number of required services for participation (Cleveland et al., 2020). Given this potential problem, we examined satisfaction of the behavioral activation intervention by assessing patients’ attitudes about participating in the group. We hypothesized that the group would be viewed positively by participants. Such a demonstration could add to the evidence base for integrated models of care in treating this underserved population.

## Methods

### Study Conditions

In this retrospective observational study, data were abstracted from the electronic health records of pregnant women (*N* = 68) receiving treatment in the Substance Abuse Treatment, Education and Prevention Program (STEPP) from February to May of 2019. STEPP is a specialty unit within the Department of Maternal Fetal Medicine at an urban academic medical center. The STEPP clinic provides integrated and co-located high-risk obstetric care, medication for opioid use disorder (MOUD), and behavioral health interventions for pregnant women with opioid use disorder. Patients were seen on a weekly basis. Obstetrical care and management of MOUD was provided by certified obstetricians and nurse practitioners. Behavioral activation groups were led by a master’s level licensed clinical social worker. A supportive therapy group for addiction recovery was also provided in the clinic. Patients in need of psychiatric medication were referred to psychiatric providers; 33 of 68 patients received psychiatric medication.

The present study focuses on the effectiveness of the behavioral health component of STEPP, which utilized Behavioral Activation for Perinatal Depression, a 12-week behavioral activation protocol devised by Gollan and Juskiewicz (Gollan & Juskiewicz, 2015). The manual is based on principles outlined in Martell et al. (2010), adapted for the perinatal context. The intervention was chosen based on evidence of its effectiveness in reducing peripartum depression (Dimidjian et al., 2017). There are also indications that behavioral activation approaches can improve substance use outcomes by enhancing positive reward behaviors and increasing social support (Daughters et al., 2018). Key components of behavioral activation therapies for depression include psychoeducation about peripartum depression; scheduling reward activities that align with personal values; detecting and preventing patterns of behavioral avoidance; and goal setting. Sessions also addressed specific peripartum challenges, such as adjusting to new caretaker roles, managing changes to routines, and dealing with barriers such as childcare and travel. Weekly group sessions were 60 min in duration. The initial session involved orientation to the group and rationale for the intervention. Each subsequent session involved review of daily activity monitoring, introduction of a new topic related to behavioral activation principles (e.g., boosting activities associated with mastery), and activity planning for the coming week. Patient-reported outcomes (PROs) were collected as part of standard care at the end of each weekly session over the course of the 12-week protocol.

### Participants

Sixty-eight pregnant or newly post-partum women with opioid use disorder participated. Table 1 provides detail on demographic and pregnancy-related variables for the sample.

**Table 1:** Demographic and pregnancy-related statistics for sample of pregnant or newly post-partum women with opioid use disorder (*N* = 68)

Variable	Descriptive statistics
Age (years)	*M* = 30 (*SD* = 4.7, range = 21–40)
Race	
White	*n* = 56 (82%)
Black	*n* = 10 (15%)
Other	*n* = 2 (3%)
Marital status	
Married	*n* = 16 (23%)
Not married	*n* = 52 (77%)
Gravidity	*M* = 2.3 (range = 0–10)
Parity	
No prior deliveries	*n* = 14 (21%)
One or more prior deliveries	*n* = 54 (79%)
Gestational age at treatment start	*M* = 24 weeks (range = 4 weeks pregnant to 7 weeks postpartum)
Gave birth during treatment	*n* = 30 (44%)

All participants were diagnosed with an opioid use disorder, with 56 participants (82%) also meeting criteria for other substance use disorders. All participants were prescribed MOUD (buprenorphine). The majority (74%) had existing psychiatric comorbidities, with 68% diagnosed with major depressive disorder and 57% with generalized anxiety disorder. Diagnoses were based on review of the patient’s medical record. A subset of patients were prescribed psychiatric medication during the course of the intervention (*n* = 33). Table 2 provides detail on psychiatric and medical comorbidities within the sample. All participants identified as cis-gender women. There were no exclusion criteria for participation in the group; all patients seen in the STEPP clinic were invited to participate.

**Table 2:** Psychiatric and medical comorbidities for sample of pregnant or newly post-partum women with opioid use disorder (*N* = 68)

Comorbidity	Frequency (%)
Opioid use disorder	68 (100)
Other substance use disorder(s)	56 (82)
Psychiatric disorder	50 (74)
Major depressive disorder	46 (68)
Generalized anxiety disorder	39 (57)
Post-traumatic stress disorder	12 (18)
Bipolar disorder	7 (10)
Schizophrenia	3 (4)
Borderline personality disorder	2 (3)
Medical disorder	24 (35)
Hepatitis C	19 (28)
Herpes simplex virus	4 (6)
Asthma	4 (6)

### Procedures

The intervention was delivered as part of routine care in STEPP from February until May of 2019. Patients completed self-report measures of depressive and anxiety symptoms at the conclusion of each weekly group session. Individuals in need of a higher level of care, including psychiatric medications, received the appropriate additional resources while participating in the group intervention.

### Measures

#### Depressive Symptoms

The Patient Health Questionnaire (PHQ-9; Kroenke et al., 2001) was used to evaluate depressive symptoms over the previous two weeks. The nine items are each scored on a Likert scale from 0 (“not at all”) to 3 (“nearly every day”). Total scores range from 0 to 27, with 5, 10, and 20 as the cutoffs for mild, moderate, and severe depression, respectively (Kroenke et al., 2001). For this sample, baseline Cronbach’s alpha was *α* = 0.88.

#### Anxiety Symptoms

The Generalized Anxiety Disorder Scale (GAD-7; Spitzer et al., 2006) was used to evaluate anxiety symptoms of GAD over the previous two weeks. The seven items are scored on a Likert scale ranging from 0 (“not at all”) to 3 (“nearly every day”). Total scores range from 0 to 21, with 5, 10, and 15 as the cutoffs for mild, moderate, and severe anxiety, respectively (Spitzer et al., 2006). For this sample, internal consistency was *α* = 0.82.

#### Participant Satisfaction

Satisfaction with the group intervention was assessed at the end of the program via an 11-item survey created by the research team. Items assessed topics such as level of support felt from the program and its leaders, hopefulness after completing the program, effectiveness of the intervention, and knowledge gained (see Table 3 for all items). Each item was scored on a 0–10 scale, with higher scores (maximum possible score 110) indicating greater satisfaction.

**Table 3:** Item wording and descriptive data for Participant Satisfaction Survey, completed by a subset of patients (*n* = 20). Each item was scored on a 0–10 scale, with higher scores indicating greater satisfaction

Item	Mean	Standard deviation	Range
1. How would you rate your level of involvement in this program?	8.0	2.3	3–10
2. How supported did you feel in this program?	8.3	1.3	6–10
3. How would you rate your hopefulness after the program?	7.9	2.3	3–10
4. To what degree did this program impact your attitude?	7.9	2.3	3–10
5. To what degree did this program impact your behavior?	7.3	2.3	3–10
6. Did you find this program acceptable (e.g., length, expectations)?	6.7	0.5	6–9
7. To what degree did you find this program ethical?	6.6	0.7	5–9
8. To what degree did you find this program effective?	6.3	1.3	3–8
9. Did the program reduce negative side effects of depression?	6.0	1.8	1–7
10. To what degree did this program increase your knowledge about coping with depression/anxiety symptoms?	6.8	0.4	6–9
11. To what degree did you feel a sense of trust with the leaders?	6.7	0.5	6–8

### Analytic Plan

All analyses were conducted in IBM SPSS Statistics 28. For each outcome (depressive symptoms, anxiety symptoms), a hierarchical linear model (HLM) was conducted to evaluate for the effect of time over the course of the intervention. The analysis included the following covariates: number of sessions attended; gestational age (weeks) upon starting intervention; comorbid psychiatric disorder (no = 0; yes = 1); current medication for depression and/or anxiety according to patient medical records (no = 0; yes = 1); age (years); race (Caucasian = 0; African American or “other” = 1); and marital status (not married = 0; married = 1). Covariates were included to help reduce the potential confounding impact of these factors on treatment results. That is, we sought to assess the extent to which changes in symptoms may have been related to key demographic and psychiatric variables.

There are a number of ways to define treatment “completion” (e.g., Castro et al., 2021; Saxon et al., 2017). Based on work by Tagalidou et al. (2019), who recommend a 70% attendance rate as qualifying for treatment completion, treatment “completers” were defined as those who attended at least 9 of 12 sessions for the present study. Within this population, there are high rates of comorbid psychological and medical conditions, which often interfere with treatment attendance. Accessing transportation to appointments is often an added barrier for these individuals. Further, many participants ((43% of total sample, 45% of non-completers)) gave birth during the course of the intervention and may have decided not to return to the intervention post-partum. Regression analyses were conducted to compare change in PHQ-9 and GAD-7 scores for treatment completers versus non-completers. Additionally, Pearson’s correlations examined the association between number of sessions attended and the change in PHQ-9 and GAD-7 scores from pre- to post-intervention. Correlations between use of psychotropic medication and change score for the relevant outcome (e.g., depression or anxiety) were also conducted.

## Results

**Fig. 1:**
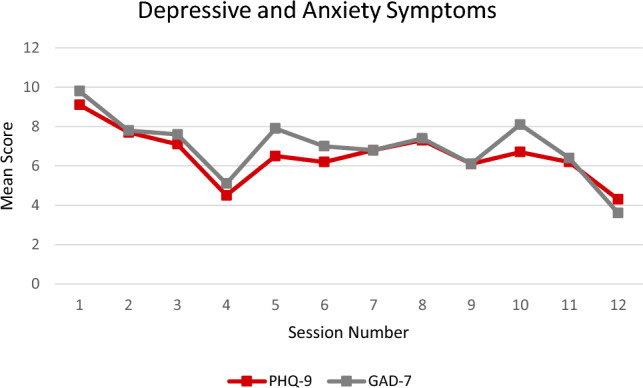
Mean PHQ-9 and GAD-7 scores over the course of 12-week intervention for pregnant or newly postpartum women with opioid use disorder

Means, standard deviations, and ranges of PHQ-9 scores and GAD-7 scores assessed at each intervention session are provided and are depicted together in Fig. 1. At initial session, mean PHQ-9 and GAD-7 scores indicate mild/moderate symptoms of depression and anxiety (mean scores 9.1 and 9.8, respectively). At the conclusion of the intervention, mean depressive and anxiety symptoms had decreased to minimal/mild severity (4.3 and 3.6, respectively).

A random intercept model was used to test the effect of time on depressive symptoms (PHQ-9). The mixed-effects model used all available data points from session 1 to session 12. Results indicate that depressive symptoms significantly decreased over time (*F*[9, 51] = 2.64, *p* = 0.014).

A random intercept model was used to test the effect of time on anxiety symptoms (GAD-7). Identical to the first model, this mixed-effects model used all available data points from session 1 to session 12. Results indicate that anxiety symptoms significantly decreased over time (*F*[9, 63] = 1.13, *p* = 0.042).

Statistical assumptions of HLM were tested. A random distribution of data points indicated that both models met the assumption of linearity. Consistent medians and interquartile ranges indicated the models met the assumption of homoscedasticity. Comparison of observed vs expected values indicated that the models met the assumption of normality.

The number of sessions attended varied between participants. The average number of sessions attended was 6.1 (*SD* = 3.1, range = 1–12). The average number of days between participants’ first and last intervention sessions was 36 (range = 0–78). There were 26 completers and 42 non-completers in the present sample. Linear regression comparing change in depressive symptoms for treatment completers vs. non-completers approached significance (R^2^ = 0.118, *F*[1, 30] = 4.03, *β* = 0.344, *p* = 0.054). The regression analysis comparing change in anxiety symptoms for completers vs. non-completers was not statistically significant (R^2^ = 0.003, *F*[1, 27] = 0.093, *β* =  − 0.059, *p* = 0.763). Of note, Pearson’s correlations demonstrated a significant, moderate association between attendance and number of points reduced on the PHQ-9 (*r* = 0.356, *p* = 0.046). The association between attendance and number of points reduced on the GAD-7 was not significant (*r* = − 0.074, *p* = 0.702). There was no significant correlation between use of psychiatric medication and reduction in depressive/anxiety symptoms (*p*s > 0.510).

The patient satisfaction survey was completed by a subset of participants (*n* = 20). Table 3 provides the mean score, standard deviation, and range for each item in the measure. The mean satisfaction score was 78.5 (SD = 10.4, range = 58–90), suggesting a general level of satisfaction with the program. Of note, the mean score on the item assessing perceived level of support by program leaders and group members was 8.3 out of 10 (SD = 1.3). The mean score on the item assessing hopefulness in one’s ability to apply learned skills and maintain progress after the program was 7.9 out of 10 (SD = 2.3).

## Discussion

The current study evaluated the effectiveness of a behavioral activation group intervention to treat depression and anxiety for peripartum women with opioid use disorder. Previous studies have shown benefits of behavioral activation for perinatal mental health problems, but have not evaluated outcomes for peripartum women with substance use disorders. Results suggest that the behavioral activation intervention was associated with reduced depression and anxiety, demonstrated by significant reductions in PHQ-9 and GAD-7 scores over the course of treatment. Moreover, results suggest that increased attendance was associated with further reductions in depressive symptoms. These findings extend research on behavioral activation for co-occurring substance use and mood problems, suggesting effectiveness for peripartum women. The intervention was also judged by participants as helpful and informative.

Importantly, this study demonstrates the effectiveness of an evidence-based psychotherapeutic intervention that was delivered in an integrated clinic providing high-risk obstetric care, substance use treatment, and mental health treatment. These encouraging results provide opportunities for clinicians and researchers to further explore the potential benefits of co-located and integrated care for this underserved population. Considerable research points at the significant barriers to accessing behavioral health treatment, which are heightened for pregnant women in need of dual-diagnosis treatment (Goodman, 2009; Raffi et al., 2021). These barriers include transportation and financial limitations, waiting lists for services, few clinics willing to treat dual-diagnosis pregnant women, and stigma directed at pregnant women with SUDs. Simply put, recommending or referring pregnant women for community treatment is likely insufficient. Instead, lowering barriers to care is essential. Providing co-located and integrated obstetric, substance use, and behavioral health care allows women to access needed treatment. Indeed, integrated care models have been associated with reduced risk for preterm birth, reduced likelihood of maternal substance use at time of delivery, and shorter infant hospitalization (Goodman et al., 2022). The results of this study add to the body of evidence supporting the effectiveness of integrated care models by pointing toward the benefits for maternal mental health. As such, the study offers a starting point for clinicians and researchers to design effective behavioral health interventions to address the important needs of this population.

While a strength of the study is its evaluation of an intervention in a real-world care setting, this setting also presents limitations. Specifically, the presence of other integrated care services and the lack of random assignment to a control condition limits causal conclusions about the impact of the behavioral activation intervention. While it is possible that other variables influenced results, the finding that increased attendance was associated with greater reduction in symptoms strongly points toward the effectiveness of the intervention. Moreover, the lack of association between psychotropic medication and symptom improvement suggests that gains were not driven by adjunct pharmacotherapy. Another limitation relates to missing data due to patient non-attendance, limiting the ability to fully assess changes in symptoms over the course of treatment. For instance, it is possible that patients stopped attending sessions due to increases in mental health symptoms, which would inflate the apparent effectiveness of the treatment. The positive association between attendance and symptom reduction suggests this is unlikely, and there were certainly many other reasons for non-attendance, importantly including giving birth. One other potential limitation relates to the clinical significance of the reduction in symptoms observed in the study. Specifically, mean depression and anxiety scores began in the mild to moderate range, and dropped to the minimal to mild range at post-treatment. While this could be interpreted as a fairly modest decrease in symptoms, it is also possible that the treatment prevented an increase in symptoms during the perinatal period, a time in which women are at increased risk for the development of depressive and anxiety disorders (Howard et al., 2014). The presence of a control condition would allow for a clearer understanding of the possible utility of the intervention in preventing a worsening of symptoms.

As noted, a randomized clinical trial comparing the peripartum behavioral activation intervention to a control condition would address several of this study’s limitations and would be a valuable next step in this line of research. Initial studies could compare the intervention to a wait-list condition or the standard practice of referral to community providers. Additionally, future studies should seek to assess patients after the conclusion of treatment, to better understand the durability of treatment gains. It would also be helpful to assess the potential impact of the treatment on obstetric and substance-related outcomes, in addition to mental health outcomes.

While future studies can add significantly to understandings of mental health and substance use treatment for peripartum women with opioid use disorder, the current study adds to the evidence supporting the efficacy of co-located, integrated care by demonstrating improvements in mental health symptoms. Importantly, the treatment was implemented in the real-world setting of a hospital-based clinic, and involved a brief, manualized intervention. The positive results of the intervention thus provide a potential blueprint for providers to reduce barriers to care and address the crucial mental health needs of peripartum women with opioid use disorder.

## Data Availability

The data that support the findings of this study are available from the corresponding author, MV, upon reasonable request.
